# Interleukin-6: the missing element of the neurocognitive deterioration in schizophrenia? The focus on genetic underpinnings, cognitive impairment and clinical manifestation

**DOI:** 10.1007/s00406-014-0533-5

**Published:** 2014-09-12

**Authors:** Dorota Frydecka, Błażej Misiak, Edyta Pawlak-Adamska, Lidia Karabon, Anna Tomkiewicz, Paweł Sedlaczek, Andrzej Kiejna, Jan Aleksander Beszłej

**Affiliations:** 1 Department of Psychiatry, Wroclaw Medical University, 10 Pasteur Street, 50-367 Wrocław, Poland; 2 Department of Genetics, Wroclaw Medical University, 1 Marcinkowski Street, 50-368 Wrocław, Poland; 3 Laboratory of Immunopathology, Department of Experimental Therapy, Institute of Immunology and Experimental Therapy, Polish Academy of Sciences, Weigla 12, 51-114 Wrocław, Poland; 4 Department of Forensic Medicine, Wroclaw Medical University, Mikulicza-Radeckiego 4, 50-368 Wrocław, Poland; 5 1st Department and Clinic of Gynaecology and Obstetrics, Wroclaw Medical University, Chałubińskiego 3, 50-368 Wrocław, Poland

**Keywords:** Interleukin-6, C-reactive protein, Gene polymorphism, Cognition, Schizophrenia, Inflammation

## Abstract

The influence of the immune system deregulation on the risk of schizophrenia is increasingly recognized. The aim of this study was to assess the influence of serum interleukin-6 (IL-6) level together with the polymorphism in its gene (*IL6* -174G/C) and high sensitivity C-reactive protein (hsCRP) levels on clinical manifestation and cognition in schizophrenia patients. We recruited 151 patients with schizophrenia and 194 healthy control subjects. Psychopathology was evaluated using Operational Criteria for Psychotic Illness checklist, Positive and Negative Syndrome Scale (PANSS) and Scales for Assessment of Positive and Negative Symptoms. Cognitive performance in schizophrenia patients was assessed using following tests: Rey Auditory Verbal Learning Test, Trail Making Test, Verbal Fluency Tests, Stroop and subscales from Wechsler Adults Intelligence Scale-R-Pl (Similarities, Digit Symbol Coding, Digit Span Forward and Backward). Serum IL-6 and hsCRP levels were significantly higher in schizophrenia patients in comparison with healthy controls. Both hsCRP and IL-6 levels were associated with insidious psychosis onset, duration of illness and chronic schizophrenia course with deterioration. After adjustment for age, education level, number of years of completed education, illness duration, total PANSS score, depression severity and chlorpromazine equivalent, there was still a positive association between IL-6 and hsCRP levels and worse cognitive performance. The *IL6* -174G/C polymorphism did not influence IL-6 level, but it was associated with the severity of positive symptoms. Our results suggest that elevated IL-6 levels may play the role in cognitive impairment and serve as potential inflammatory biomarker of deterioration in schizophrenia.

## Introduction

Interleukin-6 (IL-6) is a multifactorial cytokine that is implicated in hematopoiesis, metabolic control, bone metabolism and nociceptive regulation [1]. Notably, IL-6 has been implicated in the pathophysiology of schizophrenia. The first meta-analysis of cytokine alterations in schizophrenia patients provided that IL-6 level is increased in this group of patients [2]. These findings are in line with animal models showing that IL-6 promotes the survival of catecholaminergic neurones as well as stimulates serotonergic and dopaminergic transmission in the hippocampus and prefrontal cortex [3]. In the recent meta-analysis by Miller et al. [4], IL-6 was found to serve as a state marker of schizophrenia, which is increased in first-episode psychosis and acutely relapsed patients, and prone to normalize with antipsychotic treatment. Interestingly, emerging evidence indicates that IL-6 level is increased already in subjects with at-risk mental state (ARMS) and might be a marker of transition from ARMS to schizophrenia [5].

There is scarcity of studies looking into clinical correlates of IL-6 alterations in schizophrenia. It has been found that treatment-resistant schizophrenia is associated with elevated level of IL-6 [6]. In addition, Ganguli et al. [7] provided a positive correlation between IL-6 level and illness duration. However, the association between plasma IL-6 level and psychopathological manifestation of schizophrenia together with cognitive functioning among this group of patients has not been investigated so far.

There are also studies reporting the association between the -174 G/C polymorphism in the IL-6 gene (*IL6*) and schizophrenia. This polymorphism lies in the promoter region that accounts for transcription induced by viruses, second messengers or other cytokines [8] and leads to decrease in the *IL6* gene expression [9]. Although one study found that the *IL6*-174 G/C gene polymorphism influences the risk of schizophrenia [10] and another study that found a trend toward a significant difference in genotype distribution and allele frequency between paranoid schizophrenia patients and healthy controls [11], other authors, including our group [12], have not confirmed this association [13–15]. There is also one study by Zakharyan et al. [10] looking at the relationship between the *IL6* -174G/C polymorphism and plasma IL-6 levels in schizophrenia patients. In this study, it was shown that the *IL6* -174G/C polymorphism is associated with increased plasma IL-6 in schizophrenia patients and constitutes a risk factor for the disorder.

Extensive evidence suggests that IL-6 may influence cognitive functioning. Studies on healthy adults indicate that IL-6 plasma level negatively correlates with semantic fluency [16], auditory recognition memory, attention, working memory, as well as executive functions [17]. Other studies performed in older subjects have reported a positive association between plasma IL-6 level and impairment in a considerable number cognitive domains including working memory [18], executive functioning [19], processing speed and attention [20], orientation, immediate verbal recall, delayed recall or psychomotor speed [21], semantic fluency and prospective memory [22]. Finally, there has been an inverse relationship between plasma IL-6 level and verbal memory in patients with depression [23].

Cognitive functioning is moderately to severely impaired in most important domains including attention, memory, reasoning and processing speed in schizophrenia patients [24]. However, cognition is sparsely addressed in studies investigating immune and inflammatory aspects of schizophrenia. There is only one study showing that a subclinical inflammation, manifested in elevated level of C-reactive protein (CRP), is associated with poorer cognitive performance [25].

Interestingly, growing evidence from clinical studies with nonsteroidal anti-inflammatory agents points to the favorable effects of immunomodulatory therapy in schizophrenia, in particular in an early stage of the disorder [26]. Moreover, it has also been shown that anti-inflammatory add-on treatment may not only have beneficial effects with respect to symptoms severity as shown by recent meta-analyses [27, 28], but in particular attenuates cognitive impairment among patients with schizophrenia [29].

This study aimed at bridging important gaps in IL-6 research in schizophrenia patients, including the association between the *IL6* -174G/C polymorphism and serum IL-6 level, the influence of IL-6 level alterations on clinical and psychopathological manifestation, as well as the relationship between IL-6 production and cognitive functioning among patients with schizophrenia. Our main hypothesis was that the G allele of the *IL6* -174G/C polymorphism could predict the higher expression of IL-6, while the higher IL-6 level could be a risk factor for progressive cognitive decline observed in schizophrenia.

## Materials and methods

### Subjects

We recruited 151 patients with schizophrenia (82 females and 69 males of mean age 37.84 ± 11.56), including individuals during a recovery from acute relapse or stable outpatients and 194 controls (91 females and 103 males of mean age 39.22 ± 11.95) (Table 1). All participants were of Caucasian origin and came from the same geographic area—Lower Silesia. Study protocol was approved by the Wroclaw Medical University Ethics Committee and all subjects gave an informed consent after the nature of the procedures had been fully explained. The study was performed in accordance with the latest version of the Declaration of Helsinki. There were following exclusion criteria in the patients group: history of traumatic brain injury, neurologic disorders, severe physical health impairments and comorbid substance addiction (with exception of nicotine). A diagnosis of schizophrenia was established by the same two senior-board psychiatrists according to DSM-IV criteria and based on individual interviews, clinical observation and medical records. Lifetime psychopathology of schizophrenia was assessed using Operational Criteria for Psychotic Illness (OPCRIT) checklist [30]. Additionally, general appraisal of the course of disorder has been conducted according to the OPCRIT guidelines dividing patients to the following categories: Single episode with good recovery, multiple episodes with good recovery between, multiple episodes with partial recovery between, continuous chronic illness and continuous chronic illness with deterioration [30]. It has been assessed based on the interpersonal, social and vocational functioning in comparison with the premorbid level of functioning. All of the patients have been medicated on the day of the assessment. The mean duration of treatment was 12.24 ± 12.24 years. The majority of patients were treated with the second generation antipsychotic drugs (14.58 % olanzapine, 27.08 % risperidone, 12.50 % quetiapine, 21.87 % clozapine, 11.45 % ziprasidone, 2.08 % aripiprazole and 2.08 % sertindole), while the rest of the patients were treated with the first generation antipsychotics (2.08 % chlorpromazine, 1.04 % perazine, 1.04 % zucklopenthixol and 4.16 % haloperidol). The mean value of daily chlorpromazine (CPZ) equivalent dose was 571.84 ± 401.98 mg/day [31].

**Table 1:** General characteristics of schizophrenia patients and healthy control subjects

Demographic and clinical variables	SCH *n* = 151	HC *n* = 194	*p* value^a^
Age (years)	37.84 (11.56)	39.22 (11.95)	0.28
Female (%)	54 %	47 %	0.30
Education (years)	12.67 (3.80)	13.67 (3.35)	0.09
Ethnicity (Caucasian)	100 %	100 %	1.00
Disease duration (years)	12.24 (9.22)	–	–
Number of previous episodes	5.47 (4.66)	–	–

*SCH* schizophrenia patients, *HC* healthy control subjects

^a^ANOVA test

All healthy controls had negative present, past and family history of psychiatric illness. Patients with schizophrenia were excluded if they had current infections, allergies, severe physical health illness, present and past history of autoimmune disorders as well as abnormal blood and urine tests such as hemoglobin, hematocrit, liver transaminases, serum electrolytes, blood urea and creatinine. Current psychopathology status, cognitive performance and the *IL6* -174 G/C polymorphism were assessed in the whole study group, while serum high sensitivity CRP (hsCRP) and IL-6 levels were measured in a randomly chosen subgroup of 88 schizophrenia patients and 88 healthy controls (Table 2).

**Table 2:** The comparison of IL-6 levels between schizophrenia patients and healthy controls with respect to distinct genotypes and alleles’ carriers of the IL6 -174 G/C polymorphism

Study group	*IL6* -174G/C genotype			*p* value^a^	*IL6* -174G/C allele carriers		*p* value^b^
	CC	GC	GG		GC + CC	GG	
SCH	1.30 (0.59–3.42) (*n* = 21)	1.50 (0.79–3.14) (*n* = 38)	1.50 (0.72–2.64) (*n* = 28)	0.81	1.40 (0.69–3.06) (*n* = 59)	1.50 (0.72–2.64) (*n* = 28)	0.93
HC	1.24 (0.90–1.60) (*n* = 22)	0.98 (0.72–1.51) (*n* = 39)	1.07 (0.72–1.51) (*n* = 27)	0.51	1.07 (0.81–1.60) (*n* = 61)	1.07 (0.72–1.51) (*n* = 27)	0.24

Median values and interquartile range of IL-6 levels are presented [pg/ml]

*n* number of patients, *SCH* schizophrenia patients, *HC* healthy control subjects

^a^Kruskal–Wallis test, ^b^ Mann–Whitney *U* test

### Assessment of psychopathology and cognitive functions

Symptoms severity on the day of assessment was evaluated using Positive and Negative Syndrome Scale (PANSS), Scale for Assessment of Positive Symptoms (SAPS) and Scale for Assessment of Negative Symptoms (SANS).

Cognitive performance in schizophrenia patients was assessed using Rey Auditory Verbal Learning Test (RAVLT) [32], Trail Making Tests (TMT-A and TMT-B) [33], Verbal Fluency Tests FAS letters [34] and Supermarket [35], Stroop test [36] as well as selected Wechsler Adults Intelligence Scale (WAIS-R-Pl) subtests: Digit Symbol Coding Test, Digit Span Forward and Backward and Similarities.

### Genotyping

Genomic DNA was obtained from peripheral blood leukocytes (from whole frozen blood) using the QIAamp DNA Blood Mini Kit (Qiagen GmbH, Hilden, Germany). The *IL6* -174G/C polymorphism was genotyped using the PCR with sequence specific primers technique (PCR-SSP) with the use of PCTYGEN kit (One Lambda, Canoga Park, USA). PCR products were visualized on 2 % agarose gel.

### IL-6 and hsCRP measurement

The serum samples of 88 schizophrenia patients and 88 healthy subjects were stored in aliquots at −80 °C. Serum concentrations were measured blinded for case and control status. Serum hsCRP was measured with the use of the C-Reactive Protein Extended Range (RCRP) method on the Dimension^®^ clinical chemistry system (Siemens Healthcare Diagnostics Inc., Newark USA). Serum levels of IL-6 were measured using a commercially available human IL-6 Immunoassay (Quantikine^®^ ELISA, R&D Systems, Inc., Minneapolis, MN), according to the manufacturer’s instructions. Manual reference data for intra-assay and inter-assay coefficients of variance is 1.6–4.2 and 3.3–6.4 %, respectively.

### Statistics

The differences in serum levels of IL-6 between subjects with different *IL6* -174G/C genotypes and allele carriers in schizophrenia patients and healthy controls were compared using Kruskal–Wallis test and Mann–Whitney *U* test. Demographic and clinical data with respect to the *IL6* -174G/C polymorphism among patients with schizophrenia were compared using ANOVA test (BMI, age, pack-year, chlorpromazine equivalent, years of education, disease duration and number of previous episodes) and χ^2^ test (gender, education, family history of schizophrenia and course of the disorder). Cognitive performance and psychopathological manifestation with respect to the *IL6* -174G/C polymorphism was compared using ANOVA test; however, in case of RAVLT (recognition subtest), Kruskal–Wallis test was used due to lack of homogeneity of variances assessed by Levene’s test. Correlations between clinical variables and cognitive functioning with serum IL-6 and hsCRP levels were assessed using Spearman’s rank correlations (Spearman’s rho). Due to multiple testing of correlations between clinical variables or cognitive functioning with serum IL-6 and hsCRP, Bonferroni correction was applied to the level of significance. The association between immune markers and cognitive functioning was performed using linear regression analysis adjusting for age, education level, number of years of completed education, illness duration, total PANSS score, depression severity and chlorpromazine equivalent. Differences were considered as statistically significant if the *p* value was <0.05. All analyses were performed using the Statistical Package for Social Sciences (SPSS) version 20.

## Results

The comparison of genotype distribution and allele frequency of the *IL6* -174G/C polymorphism between schizophrenia patients and healthy controls was presented in our previous paper [12]. There was no significant association between the *IL6* -174G/C polymorphism and the risk of schizophrenia.

Serum IL-6 was significantly higher in schizophrenia patients in comparison with healthy controls (median: 1.4 ± 2.23 and 1.07 ± 0.77, respectively, *p* = 0.049). Similarly, serum hsCRP was significantly higher in schizophrenia patients in comparison with healthy controls (median: 3.30 ± 3.7 and 2.30 ± 1.6, respectively, *p* < 0.001). There was no association between the *IL6* -174G/C polymorphism and serum IL-6 in schizophrenia patients and healthy controls (Table 2). Similarly, there was no significant difference in demographics and general clinical variables with respect to the *IL6* -174G/C polymorphism in schizophrenia patients (Table 3). We found no significant difference between the variables that may influence the relationship between the *IL6* -174G/C polymorphism and IL-6 levels, such as age, gender, chlorpromazine equivalent, illness duration, cigarette smoking and BMI across the *IL6* -174G/C genotypes (Table 3).

**Table 3:** Demographic and clinical characteristics of schizophrenia patients with respect to the *IL6* -174G/C polymorphism

Demographic and clinical variables	The *IL6* -174G/C genotype			*p* value^a^
	CC (*n* = 39)	GC (*n* = 72)	GG (*n* = 40)	
Age (years)	39.90 (10.7)	37.47 (9.7)	40.20 (10.6)	0.18
Female (%)	52 %	43 %	44 %	0.56^b^
Education (%)				0.68^b^
Primary	13 %	7 %	8 %	
Vocational	22 %	20 %	20 %	
Secondary	52 %	47 %	60 %	
Higher	13 %	26 %	12 %	
Education (years)	12.30 (3.77)	12.89 (3.95)	11.92 (4.50)	0.62
Disease duration (years)	12.77 (8.73)	11.32 (9.94)	13.13 (9.10)	0.63
Number of previous episodes	5.38 (3.12)	4.64 (3.87)	6.94 (6.42)	0.25
Family history of schizophrenia in first or second degree relative (%)	8.06 %	19.36 %	6.45 %	0.30^b^
Chronic course of disorder with deterioration (%)	7.26 %	16.93 %	8.06 %	0.81^b^
Chlorpromazine equivalent	530.25 (299.04)	604.40 (418.13)	557.53 (469.30)	0.79
Body mass index (BMI) (kg/m^2^)	26.01 (3.52)	27.11 (5.10)	28.63 (8.73)	0.46
Pack-year Index^c^	6.55 (9.48)	10.51 (16.80)	10.75 (10.24)	0.69

Mean and standard deviation values are presented

*n* number of patients

^a^ANOVA test

^b^χ^2^ test

^c^Pack-year Index was calculated as the number of cigarettes packs per 1 day multiplied by number of years smoked and divided by 20

Finally, this polymorphism was not associated with the psychopathological manifestation assessed with OPCRIT (data not shown) and psychopathological scales nor with cognitive functioning (Table 3). However, the comparison of positive symptoms severity assessed using SAPS showed significant differences between homozygotes (*p* = 0.027) and trend level differences when assessed using PANSS (*p* = 0.07). Indeed, the -174GG homozygotes had higher severity of positive symptoms in comparison with the -174CC patients (data not shown).

Serum IL-6 positively correlated with hsCRP in schizophrenia patients (*r* = 0.57, *p* < 0.001) and healthy controls (*r* = 0.52, *p* < 0.001). There was no significant correlation between age and hsCRP or IL-6 levels neither in schizophrenia patients (*r* = 0.11, *p* = 0.31 and *r* = 0.26, *p* = 0.22, respectively) nor in healthy controls (*r* = 0.054, *p* = 0.60 and *r* = 0.11, *p* = 0.39).

Correlations between hsCRP or IL-6 levels and clinical variables in schizophrenia subjects are provided in Table 4. Indeed, hsCRP level was negatively associated with the scores of the majority of RAVLT subtests. However, there was a positive correlation between hsCRP level and chlorpromazine equivalent (*r* = 0.32, *p* = 0.009).

**Table 4:** Cognitive performance and psychopathological manifestation with respect to the *IL6*-174G/C polymorphism

	the *IL6* -174G/C genotype			*p* value^a^
	CC (*n* = 39)	GC (*n* = 72)	GG (*n* = 40)	
PANSS—positive symptoms	17, 17.99 (6.23)	18, 19.74 (7.72)	23, 21.76 (5.95)	0.24
PANSS—negative symptoms	24, 23.09 (7.57)	21, 21.89 (8.52)	24, 25.28 (7.85)	0.28
PANSS—general symptoms	39, 39.74 (7.89)	38, 39.79 (11.28)	43, 43.64 (11.66)	0.53
PANSS—total score	82, 80.78 (17.68)	75, 81.42 (24.96)	87, 90.22 (23.65)	0.36
PANSS—depression item	1, 1.77 (1.10)	1, 1.72 (1.01)	1, 1.70 (1.17)	0.97
SAPS	26, 19.71 (15.93)	25, 31.32 (21,47)	37, 38.48 (20.37)	0.11
SANS	37, 41.39 (19.44)	31, 36.53 (21.39)	45, 47.64 (21.11)	0.12
TMT, part A	45, 48.80 (27.5)	41, 50.46 (48.3)	42, 39.98 (29.0)	0.98
TMT, part B	109, 136.58 (99.0)	101, 140.17 (113.7)	110, 101.73 (79.3)	0.95
Stroop, congruent	37, 40.71 (12.9)	40, 37.08 (22.0)	41, 40.53 (17.7)	0.49
Stroop, incongruent	81.50, 87.75 (35.8)	72, 80.04 (35.6)	75, 81.33 (39.7)	0.70
RAVLT, first immediate recall	4, 3.25 (1.93)	4.50, 4.35 (1.87)	4, 4.55 (2.38)	0.79
RAVLT, second immediate recall	6, 6.39 (2.59)	7, 6.32 (2.45)	6, 7.39 (2.62)	0.78
RAVLT, third immediate recall	8, 7.39 (2.62)	7, 7.14 (2.43)	8, 7.27 (2.98)	0.88
RAVLT, fourth immediate recall	9, 8.30 (2.98)	8, 8.43 (2.63)	9, 8.95 (3,03)	0.93
RAVLT, fifth immediate recall	9, 8.78 (3.60)	9, 8,84 (2.30)	9, 9.45 (3.00)	0.70
RAVLT, inference	8, 7.32 (3,03)	7, 7.43 (2.91)	7, 7.60 (2.99)	0.96
RAVLT, delayed recall	7, 6.00 (3.49)	6.5, 6.65 (2.44)	6, 6.84 (3.10)	0.74
RAVLT, recognition	11, 9.41 (4.68)	10, 9.90 (3.48)	10, 9.24 (3.60)	0.48^b^
Verbal fluency, F words	5, 5.91(3.76)	7, 7.22 (3.70)	6, 5.4 3 (4.16)	0.29
Verbal fluency, A words	5, 6.13 (3.47)	6, 7.35 (3.85)	6, 5.41 (3.30)	0.11
Verbal fluency, S words	8, 8.52 (4.71)	9, 8.19 (3.25)	7, 7.91 (3.38)	0.38
Verbal fluency, K words	10, 10.43 (4.94)	12, 12.32 (4.61)	10, 10.59 (4.23)	0.18
Verbal fluency, supermarket	16, 17.52 (7.98)	15, 15.70 (5.85)	13, 15.14 (7.19)	0.35
Forward digit span	6, 6.14 (1.78)	6, 6,20 (1.96)	6, 6.08 (1.89)	0.90
Backward digit span	5, 5.23 (2.47)	5, 5.23 (2.04)	5, 5.04 (2.30)	0.99
Digit symbols coding	37.50, 40.73 (15.81)	34, 35.78 (12.49)	37, 36.88 (14.59)	0.37
Similarities	14, 14.39 (5.33)	14, 16.00 (5.41)	15, 16.55 (3.76)	0.47

*n* number of patients

Median, mean and standard deviation values are presented

^a^ANOVA test, ^b^ Kruskal–Wallis test

Serum level of IL-6 was correlated with worse cognitive performance on TMT-A (*r* = 0.38, *p* = 0.001) and TMT-B (*r* = 0.48, *p* = 0.001), Stroop test (congruent) (*r* = 0.27, *p* = 0.024) and Stroop test (incongruent) (*r* = 0.34, *p* = 0.005), Verbal Fluency (FAS total score) (*r* = −0.31, *p* = 0.011), Forward Digit Span test (*r* = −0.27, *p* = 0.026) and Digit Symbol Coding test (*r* = −0.45, *p* < 0.001). Both IL-6 and hsCRP serum levels were associated with worse performance on the majority of RAVLT subtests (Table 5).

**Table 5:** Correlations between clinical, demographic and cognitive correlates with plasma hsCRP and IL-6 levels in schizophrenia patients

Clinical correlates	hsCRP	IL-6
*Psychopathology*		
PANSS—positive symptoms subscale	*r* = −0.14, *p* = 0.20	*r* = −0.05, *p* = 0.66
PANSS—negative symptoms subscale	*r* = 0.01, *p* = 0.94	*r* = 0.15, *p* = 0.23
PANSS—general symptoms subscale	*r* = −0.12, *p* = 0.28	*r* = −0.04, *p* = 0.73
PANSS—depression item score	*r* = −0.06, *p* = 0.60	*r* = 0.19, *p* = 0.10
SAPS	*r* = −0.03, *p* = 0.78	*r* = −0.05, *p* = 0.66
SANS	*r* = −0.05, *p* = 0.67	*r* = 0.15, *p* = 0.22
*Cognitive tasks*		
TMT, part A	*r* = 0.09, *p* = 0.39	*r* = 0.38, ***p*** **=** 0.001*
TMT, part B	*r* = 0.19, *p* = 0.09	*r* = 0.48, ***p*** **=** 0.001*
TMT, part A- TMT, part B	*r* = 0.19, *p* = 0.09	*r* = 0.34, ***p*** **=** **0.005**
Stroop, congruent	*r* = 0.06, *p* = 0.58	*r* = 0.27, ***p*** **=** **0.024**
Stroop, incongruent	*r* = 0.17, *p* = 0.13	*r* = 0.34, ***p*** **=** **0.005**
RAVLT, first immediate recall	*r* = −0.38, ***p*** ***<*** 0.001*	*r* = −0.37, ***p*** **=** **0.002**
RAVLT, second immediate recall	*r* = −0.33, ***p*** ***<*** 0.001*	*r* = −0.31, ***p*** **=** **0.009**
RAVLT, third immediate recall	*r* = −0.32, ***p*** **=** **0.003**	*r* = −0.40, ***p*** **=** 0.001*
RAVLT, fourth immediate recall	*r* = −0.34, ***p*** **=** **0.002**	*r* = −0.39, ***p*** **=** 0.001*
RAVLT, fifth immediate recall	*r* = −0.29, ***p*** **=** **0.007**	*r* = −0.37, ***p*** **=** 0.001*
RAVLT, inference	*r* = −0.15, *p* = 0.170	*r* = −0.25, ***p*** **=** **0.037**
RAVLT, delayed recall	*r* = −0.23, ***p*** **=** **0.049**	*r* = −0.28, ***p*** **=** **0.024**
RAVLT, recognition	*r* = −0.33, ***p*** **=** **0.004**	*r* = −0.36, ***p*** **=** **0.003**
Verbal fluency, F words	*r* = 0.16, *p* = 0.162	*r* = −0.23, *p* = 0.059
Verbal fluency, A words	*r* = −0.01, *p* = 0.927	*r* = −0.29, ***p*** **=** **0.014**
Verbal fluency, S words	*r* = 0.16, *p* = 0.163	*r* = −0.17, *p* = 0.156
Verbal fluency, FAS total score	*r* = 0.11, *p* = 0.329	*r* = −0.31, ***p*** **=** **0.011**
Verbal fluency, K words	*r* = 0.07, *p* = 0.550	*r* = −0.22, *p* = 0.087
Verbal fluency, supermarket	*r* = 0.11, *p* = 0.343	*r* = 0.13 *p* = 0.289
Forward digit span	*r* = −0.01, *p* = 0.920	*r* = −0.27, ***p*** **=** **0.026**
Backward digit span	*r* = −0.04, *p* = 0.730	*r* = −0.17, *p* = 0.164
Digit symbol coding	*r* = −0.19, *p* = 0.098	*r* = −0.45, ***p*** ***<*** 0.001*
Similarities	*r* = −0.02, *p* = 0.842	*r* = 0.48, *p* = 0.149
*Demographic and clinical correlates*		
Age	*r* = 0.11, *p* = 0.31	*r* = 0.26, *p* = 0.220
BMI	*r* = 0.12, *p* = 0.505	*r* = 0.26, *p* = 0.062
Pack-year index	*r* = 0.05, *p* = 0.781	*r* = 0.09, *p* = 0.523
Chlorpromazine equivalent	*r* = 0.32, ***p*** **=** **0.009**	*r* = 0.57, ***p*** ***<*** 0.001*
Illness duration	*r* = 0.19, *p* = 0.073	*r* = 0.30, ***p*** **=** **0.008**

Significant associations were marked in bold characters (*p* value <0.05, 2-tailed)

* Significant correlations after the application of Bonferroni correction (*p* < 0.00147)

The correlations of IL-6 level with cognitive performance in TMT tasks, RAVLT subtests (third, fourth and fifth immediate recall) and Digit Symbol Coding test, as well as the correlation of hsCRP with RAVLT subtests (first and second immediate recall) remained significant after adjustment for multiple testing (*p* < 0.00147) (Table 5).

**Fig. 1:**
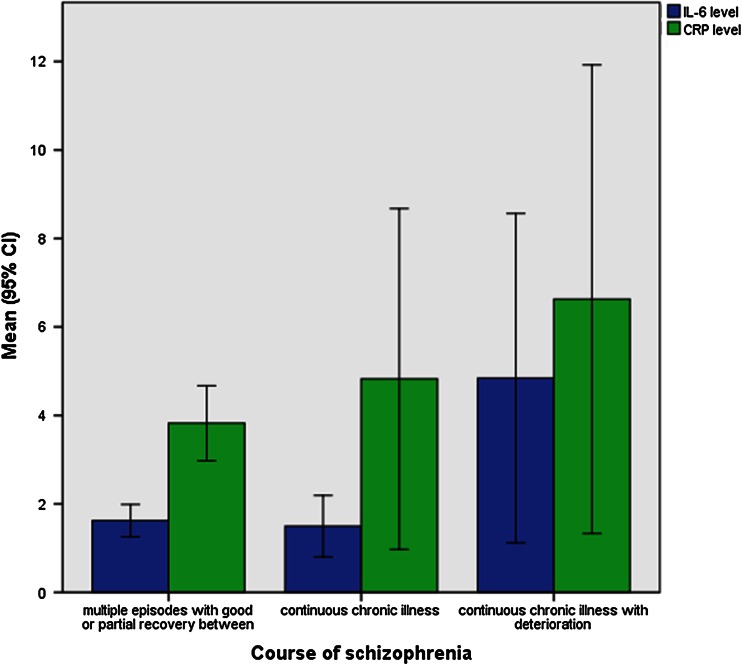
The comparison of hsCRP and IL-6 (*p* > 0.05 and *p* = 0.01, respectively) levels among patients with different course of schizophrenia (multiple episodes with good or partial recovery between episodes, continuous chronic illness, continuous chronic illness with deterioration)

Chlorpromazine equivalent was significantly associated with higher IL-6 level (*r* = 0.57, *p* < 0.001), even after the application of Bonferroni correction (*p* < 0.00147). Interestingly, longer illness duration predicted higher IL-6 levels (*r* = 0.30, *p* = 0.008). Additionally, there was a higher level of serum IL-6 among patients with schizophrenia characterized by chronic course with deterioration in comparison with the schizophrenic patients with multiple episodes with good or partial recovery or even with chronic course; however, without marked deterioration of functioning (*p* = 0.01) (Fig. 1).

After adjustment for age, education level, number of years of completed education, illness duration, total PANSS score, depression severity in PANSS and chlorpromazine equivalent, there was a trend level association of hsCRP and statistically significant association of IL-6 with lower scores on immediate recall span assessed by RAVLT (total score summed across 1st through fifth trial) (*β* = −0.13 95 %CI −0.26–0.01, *p* = 0.06; *β* = −0.05, 95 %CI −0.10–0.01, *p* = 0.04, respectively). Additionally, higher IL-6 levels predicted worse cognitive performance on Digit Symbols Coding test (*β* = −0.62, 95 %CI −0.11–0.16, *p* = 0.009) (Table 6).

**Table 6:** Associations between cognitive tests score and serum IL-6 and hsCRP after adjustment for age, education level, number of years of completed education, illness duration, total positive and negative syndrome scale (PANSS) score

Cognitive tasks	hsCRP	*p*	IL6	*p*
RAVLT immediate recall—total score	−0.13 (−0.26–0.01)	0.06	−0.05 (−0.10–0.01)	**0.04**
RAVLT, distraction	−0.26 (−0.81–0.28)	0.34	−0.093 (−0.37–0.12)	0.38
RAVLT, delayed recall	−0.13 (−0.60–0.05)	0.56	−0.10 (0.−0.32–0.12)	0.37
RAVLT, recognition	−0.15 (−0.48 to −0.18)	0.36	−0.09 (−0.26–0.08)	0.27
Digit symbols coding	−0.58 (−0.19–0.75)	0.39	−0.62 (−0.11–0.16)	**0.009**

Significant associations were marked in bold characters (*p* value <0.05, 2-tailed)

## Discussion

The influence of the immune system deregulation on the risk of schizophrenia has been reported for years now; however, the association between CRP and IL-6 with schizophrenia course and symptomatology, as well as with cognitive functioning has not been shown so far. We have found that elevated IL-6 level is associated with schizophrenia, that is in line with the results of previous meta-analyses [2, 4]. We have not confirmed the influence of the *IL6* -174G/C polymorphism on IL-6 level in patients with schizophrenia that was shown in one study [10]. Discrepancies between these results may be due to the fact that the participants of our study were relatively younger (38.99 ± 10.2 vs. 46 ± 9.8 years), with earlier age of psychosis onset (25.20 ± 6.7 vs. 24.3 ± 8.1 years) and different ethnicities (Polish vs. Armenian). Finally, in our study we have controlled for the variables that may influence the relationship between the *IL6* -174G/C polymorphism and IL-6 levels, such as age, gender, chlorpromazine equivalent, illness duration, cigarette smoking and BMI.

We have not shown the influence of the *IL6* -174G/C polymorphism on the mode of onset, on the course of the disorder or on any single symptom described by OPCRIT checklist. However, -174GG homozygotes had significantly higher severity of positive symptoms in comparison with -174CC homozygotes. Interestingly, there was an increase in the level of positive symptoms with each −174G allele. So far, there is one report showing the association between 3′-UTR *IL*-*6* gene polymorphism with the positive symptom dimension in schizophrenia [37]. We have not found any correlation between serum IL-6 level and schizophrenia psychopathology. Previous studies have revealed a positive relationship between plasma IL-6 and the severity of positive symptoms in subjects with ARMS [5] and veterans with schizophrenia [38]. There is also one study showing a positive correlation between serum IL-6 and the severity of negative symptoms in drug-naïve schizophrenia males [39]. However, the vast majority of studies have not confirmed these findings [2, 4].

To the best of our knowledge, this is the first study investigating the influence of the *IL6* -174G/C polymorphism together with serum IL-6 and hsCRP levels on cognition in schizophrenia patients. We have found no significant differences in cognitive performance with respect to the *IL6* -174G/C polymorphism. However, we have found that higher IL-6 level is associated with impairment in cognitive functioning in terms of visual attention (Stroop test, TMT-A), visuomotor processing speed (Digit Symbol Coding, TMT-A), memory (Digit Span Forward, RAVLT), semantic memory (Verbal Fluency), working memory and task-switching ability (TMT-B), as well as executive control function (the difference between TMT-B and TMT-A score). In turn, hsCRP level was associated with worse verbal memory performance (RAVLT). Importantly, the correlations of IL-6 level with visual attention, task-switching ability (TMT-A and TMT-B), visuomotor processing speed (TMT-A and Digit Symbol Coding), memory performance (RAVLT) and the correlation of hsCRP with memory performance (RAVLT) remained significant after the application of restrictive Bonferroni correction. After adjustment for possible confounders, such as age, education, illness duration, psychopathology and antipsychotic treatment, both hsCRP and IL-6 were related to worse cognitive performance with respect to immediate memory span (RAVLT learning summary score), while IL-6 level was additionally associated with lower score on visuomotor processing speed (Digit Symbol Coding task).

Notably, RAVLT learning summary score is widely used and has good test–retest reliability and has been shown to discriminate memory impairment from memory intact patients [40]. Lower RAVLT scores in patients with schizophrenia have been reported repeatedly both in drug naive [41], as well as in patients during long-term antipsychotic treatment [42]; however, our study is the first to show the association of the poorer performance on RAVLT with immune biomarkers. So far, a positive association between immediate memory impairment and IL-6 level has been reported in other patients, including older people [43], middle-aged adults [16] and patients with recurrent major depressive disorder [23].

Digit Symbol Coding task has been reported to be a measure differentiating schizophrenia patients from healthy controls, low- and high-risk relatives of schizophrenia patients from healthy controls and unaffected relatives from schizophrenia subjects [44]. Apart from its discriminative value, Digit Symbol Coding task may predict poor outcome and functional disability in schizophrenia [44, 45]. Furthermore, IL-6 level has been found to predict worse cognitive performance in Digit Symbol Coding test in the elderly [46] and type 2 diabetes elders [47].

Our results confirm reports from previous studies showing that CRP level is increased in schizophrenia patients [48, 49], including drug-naïve individuals [50]. The influence of CRP level on cognition, reported in our study, corresponds with the results obtained by Dickerson and colleagues [25, 51]. Authors found that CRP level negatively correlates with the overall cognitive functioning assessed by Repeatable Battery for the Assessment of Neuropsychological Status (RBANS) in schizophrenia patients [52], as well as with total cognitive performance and specific RBANS domains including attention, immediate memory and language in bipolar disorder patients [53]. Notably, Dickerson et al. [25] also failed to find a significant correlation between CRP level and psychopathology evaluated in PANSS, although the previous study by Fan et al. [54] indicated that CRP level is associated with higher severity of negative symptoms, general psychopathology and total PANSS score. We have found a positive correlation between IL-6 level and illness duration along with significantly higher levels of IL-6 in schizophrenia patients with chronic course of the disorder with deterioration. These findings corroborate previous studies showing the positive association between IL-6 and illness duration [7, 39] and higher IL-6 in patients with deficit schizophrenia [50]. Furthermore, it has been found that IL-6 level positively correlates with duration of untreated psychosis [55]. Together with the fact that we have found the relationship between higher IL-6 levels and cognitive decline in schizophrenia, it could be inferred that IL-6 may serve as a potential inflammatory biomarker accompanying neurodegenerative processes leading to cognitive deterioration in schizophrenia.

Notably, our study has some limitations that should be addressed. A possible limitation is a small sample size. However, our sample size is similar to that from the only study looking into correlations between the *IL6* -174G/C polymorphism and IL-6 level in schizophrenia patients from Armenia  [10]. In addition, the number of patients included in our study is comparable to the sample sizes that are commonly chosen for the studies on serum cytokines levels and cognitive functions in psychiatric disorders. Another limitation is a clinical heterogeneity of the patients included in the study (inpatients during the recovery from acute psychosis and stable outpatients). However, this approach allowed us to analyze cognitive impairments with regard to the wider spectrum of psychopathology severity and underlying subthreshold inflammation. Finally, we have not assessed cognitive functioning in healthy controls. Therefore, we cannot determine as to whether obtained correlations between IL-6 level and cognitive performance are specific for schizophrenia patients. Nevertheless, our results indicate that elevated IL-6 level may play additional role in the development of cognitive deficits described in schizophrenia and may further contribute to deterioration observed in the course of the disorder.

Our results indicate that elevated IL-6 in schizophrenia patients does not occur due to genetic variation in its gene. However, the *IL6* -174G/C polymorphism may affect the severity of positive symptoms. In turn, cognitive impairments observed in schizophrenia might be the consequence of subclinical inflammation manifested in elevated hsCRP and IL-6 serum levels. Correlations between serum IL-6 level, illness duration and course of the disorder are in line with the notion that schizophrenia is a progressive disorder with low-grade inflammation, underlying its pathophysiology followed by cognitive decline.
